# Multi-omics integrated analyzed the origin of intrahepatic mucinous cholangiocarcinoma: a case report

**DOI:** 10.3389/fonc.2023.1175707

**Published:** 2023-07-21

**Authors:** Xiaokang Zeng, Huohui Ou, Chong Zeng, Qingbo Liu, Weidong Wang, Jie Yao

**Affiliations:** ^1^ Medical Research Center, Shunde Hospital, Southern Medical University, Foshan, China; ^2^ Department of Hepatobiliary Surgery, Shunde Hospital, Southern Medical University, Foshan, China; ^3^ Department of Laboratory Medicine, Shunde Hospital, Southern Medical University, Foshan, China

**Keywords:** intrahepatic mucinous cholangiocarcinoma, intrahepatic cholangiocarcinoma, whole exome sequencing, differentially expressed genes, mucin

## Abstract

Intrahepatic mucinous cholangiocarcinoma (IMCC) is a rare subtype of intrahepatic cholangiocarcinoma (IHCC). Limited data describe the genetic characteristics of IMCC and insights on its pathogenesis are lacking. Here, we employed a multi-omics approach to analyze somatic mutations, transcriptome, proteome and metabolome of tumor tissue obtained from a case of IMCC in order to clarify the pathogenesis of IMCC. A total of 54 somatic mutations were detected, including a G12D mutation in KRAS that is likely to be involved in the onset of IMCC. The genes consistently up-regulated at the transcription level and in the proteome were enriched for mucin and mucopolysaccharide biosynthesis, for cell cycle functions and for inflammatory signaling pathways. The consistently down-regulated genes were enriched in bile synthesis and fatty acid metabolism pathways. Further multi-omics analysis found that mucin synthesis by MUC4 and MUC16 was elevated by up-regulated expression of mesothelin (MSLN). Moreover, transcription factor ONECUT3 was identified that possibly activates the transcription of mucin and mucopolysaccharide biosynthesis in IMCC.

## Introduction

Intrahepatic mucinous cholangiocarcinoma (IMCC) is characterized by the secretion of large amounts of extracellular mucus. It is an extremely rare subtype of cholangiocarcinoma, accounting for about 10% of intrahepatic chlolangiocarcinoma (IHCC) cases (1). The incidence of IMCC is highest in Southeast Asia (71.3 cases per 100000 person‐years), particularly in Thailand (more than 80 cases per 100000 person‐years) (2). The previously published literature revealed that this subtype is notorious for rapid growth, widespread metastasis, and poor prognosis than the conventional iCCA (3). Currently, the diagnosis of IMCC mainly relies on imaging methods, and immunochemistry (4). However, there are currently no tumor markers for early diagnosis of IMCC. We hope to identify possible mechanisms and early biomarkers of IMCC at the genetic level through this case. To provide theoretical basis for revealing the pathogenesis of IMCC.

Multi-omics studies include the characterization of genome mutations, copy number variation, transcriptomics, proteomics and metabolomics, which in combination can reveal comprehensive and accurate molecular mechanisms of a given cancer type (5, 6). When this was applied to IHCC, most of the mutant genes identified were tumor suppressor genes or oncogenes, such as TP53, KRAS, BAP1 (7). However, a multi-omics analysis for IMCC has not yet been reported. Therefore, we employed multi-omics analysis to a single case of IMCC in an attempt to discover molecular markers that can be used for early diagnosis.

## Materials and methods

Please see the Supplementary section.

## Case presentation

### Case description

A 62-year-old female patient presented to the hospital complaining of epigastric pain. Enhanced CT scans confirmed the presence of liver space and the diagnose of primary liver cancer (**Supplementary Figure 1A**). After surgery, the extracted tissue was results of H&E staining showed that the nuclei of tumorous tissue were of different sizes and the cells were irregularly arranged (**Supplementary Figures 1B, C**). The results of immunohistochemistry showed that the markers CAE, CK19, and CK5/6 were all positive (**Supplementary Figures 1D–I**). The periodic acid-Schiff staining (PAS) indicated presence of multiple mucinous polysaccharides in the tissue (**Supplementary Figures 1J, K**). Based on these pathological results, the patient was diagnosed with IMCC.

### Genome variation detected in the IMCC tissue

The somatic mutations demonstrated by whole exome sequencing (WES), showed that the IMCC had 54 somatic mutations. After comparison we found that 2 of the 54 genes were oncogenes and 2 were tumor suppressor genes (**Supplementary Figure 2A**). However, the expression levels of RNA and protein were not consistent (**Supplementary Figures 2B, C**). This indicated that the change in the expression of somatic mutant genes was not a likely cause of IMCC. We next compared the obtained mutant genes in the IMCC case with those reported for IHCC and squamous cell intrahepatic cholangiocarcinoma (SCC-IHCC). This identified 37 mutant genes that were specific for IMCC (**Supplementary Table 1**). IMCC and the other two subtypes shared 17 mutant genes, of which 16 were shared with IHCC, while 1 was shared with SCC-IHCC (**Supplementary Figures 2D, E**). We found 54 somatic mutation genes in IMCC sample, two of which are oncogene and two of which are tumor suppressor genes. The two oncogenes are KRAS and IRS1. It is worth noting that the mutation of the oncogene KRAS exists in both IMCC and IHCC. Sequenced the KRAS gene identified the mutation locus in G12D (rs121913529) of IMCC (**Supplementary Figure 2F**). The mutation of KRAS at G12D locus is the 12th amino acid mutation (glycine to aspartate), which is a widely reported oncogenic driving gene (8, 9).

### Transcriptome characteristics of IMCC

**Figure 1:**
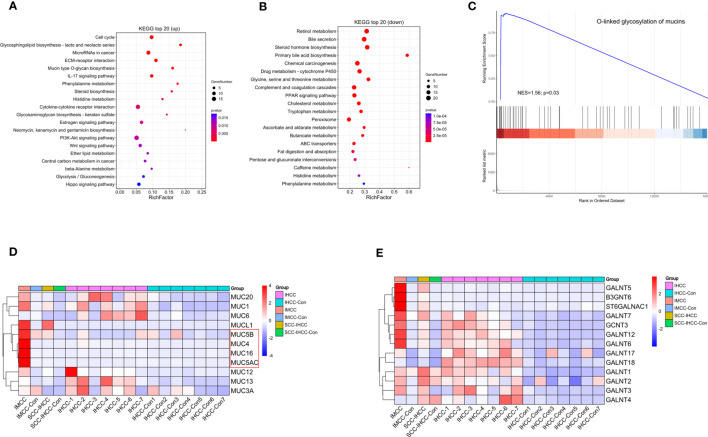
The expression of genes involved in mucin and mucopolysaccharide biosynthesis is up-regulating in IMCC. KEGG analysis identified the main enriched signaling pathways of up-regulated genes **(A)** and of down-regulated genes **(B)** in IMCC. Gene enrichment analysis shows the activation of O-linked glycosylated mucins **(C)**. Heatmaps of expression levels in IMCC, IHCC and SSC-IHCC of mucin **(D)** and of mucopolysaccharide synthesis-related genes **(E)**.

Up-regulated genes were found to be significantly enriched in mucin and mucopolysaccharide synthesis, cell cycle and inflammation-related pathways (**Figure 1A**). Increased gene expression in mucin and mucopolysaccharide biosynthesis, cell cycle, and inflammatory pathways may increase the synthesis of mucus and mucopolysaccharides in IMCC tissue, accelerate cell cycle, and increase inflammation levels. Ultimately, it led to the appearance of high mucus content in IMCC. Previous studies have also demonstrated elevated expression of mucin related genes, cell cycle, and inflammation related genes in IMCC (10, 11). The down-regulated genes were mainly enriched in pathways of bile synthesis and secretion and fat digestion (**Figure 1B**). The downregulation of gene expression in bile synthesis and fatty acid metabolism pathways suggests a decrease in bile synthesis ability in IMCC tissue, which in turn leads to a decrease in fatty acid metabolism ability. This may be one of the mechanisms by which IMCC occurs. Previous studies also support our speculation (12, 13). A Gene Set Enrichment Analysis (GSEA) was also performed, which showed that oxygen-linked mucin glycosylation was activated in IMCC (**Figure 1C**). We compared the expression of these genes in different subtypes of cholangiocarcinomas. Except for MUC1, MUC6 (partially positive in IHCC) and MUCL1 (positive in SCC-IHCC), MUC5B, MUC4, MUC16, and MUC5AC were all specifically highly expressed in IMCC (**Figure 1D**). Among the genes related to mucopolysaccharide biosynthesis, GALNT5, B3GNT6, and ST6GALNAC1 were expressed in SCC-IHCC, but they were specifically highly expressed in IMCC (**Figure 1E**). These results showed that the mucinous phenotype of IMCC is related to a high expression of mucin and mucopolysaccharide-related genes.

### Proteomics and metabolomics characteristics of IMCC

**Figure 2:**
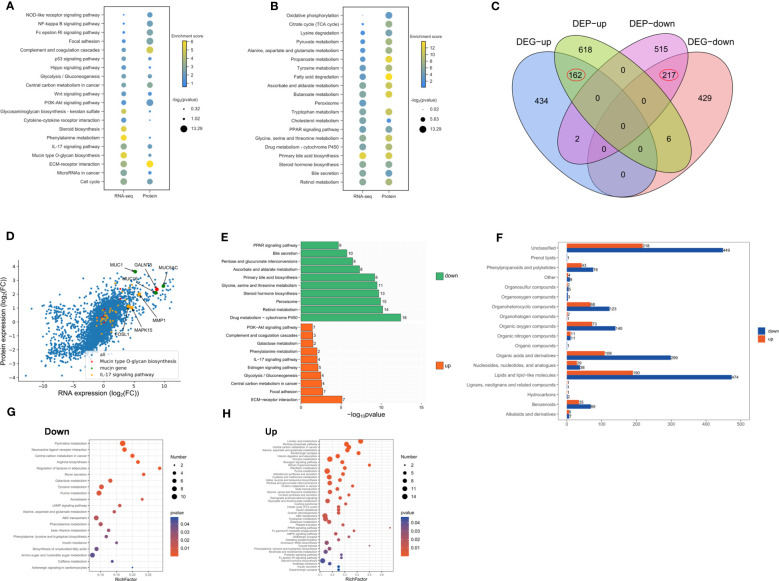
Up-regulated proteins of IMCC are mainly enriched in inflammation and cell cycle signaling pathways, while down-regulated proteins are mainly enriched in the regulation of bile acid synthesis. Dotplots show consistent KEGG enrichment in transcriptome and proteome of upregulated **(A)** and of downregulated **(B)** genes in IMCC. Venn diagram of RNA and protein of the up- and down-regulated genes **(C)**. Scatter plot of gene and protein expression to illustrate their consistency in IMCC **(D)**. Bar graph showing the enrichment of signaling pathways of genes or proteins that are jointly up-regulated or down-regulated in IMCC **(E)**. The different metabolites identified in IMCC cancer tissue and adjacent tissue **(F)**. The bubble charts show the enriched pathways of differential metabolites in IMCC **(G, H)**.

We identified up-regulated and down-regulated proteins through proteome sequencing, in order to identify significantly enriched signaling pathways. We found that the up-regulated proteins were mainly enriched in extracellular matrix receptor interactions, cell cycle and inflammation-related signaling pathways (**Figure 2A**). The down-regulated proteins were mainly enriched in the synthesis and secretion of bile, retinol metabolism, and amino acid metabolism-related signaling pathways (**Figure 2B**). A total of 162 genes were identified as up-regulated and 217 genes were down-regulated both in RNA and protein levels (**Figure 2C**). The expression of RNA and protein was visualized by scatter plots, which produced a good linear relation. The highly expressed genes were mainly enriched in mucin synthesis, mucopolysaccharide and the IL-17 signaling pathway (**Figure 2D**). The genes both up-regulated in RNA and protein were enriched in pathways such as extracellular matrix receptor interaction, IL-17 signaling and mucin secretion, while down-regulated genes were mainly enriched in bile synthesis and secretion, retinol metabolism and amino acid metabolism (**Figure 2E**). We further explored the changes of metabolites in IMCC, showing that the number of metabolites in IMCC was decreased (**Figure 2F**). The metabolites reduced in IMCC were mainly fat or fat-like substances, organic acids and their derivatives. This indicated that fatty acid metabolism in IMCC patients is hindered (**Figure 2G**). The up-regulated metabolites in IMCC were enriched in fat cell lipid catabolism pathways (**Figure 2H**). These results suggest that DNA synthesis and sugar metabolism are enhanced in IMCC, while the metabolism of linoleic acid, amino acids is weakened.

### Multi-omics comprehensive analysis of IMCC

**Figure 3:**
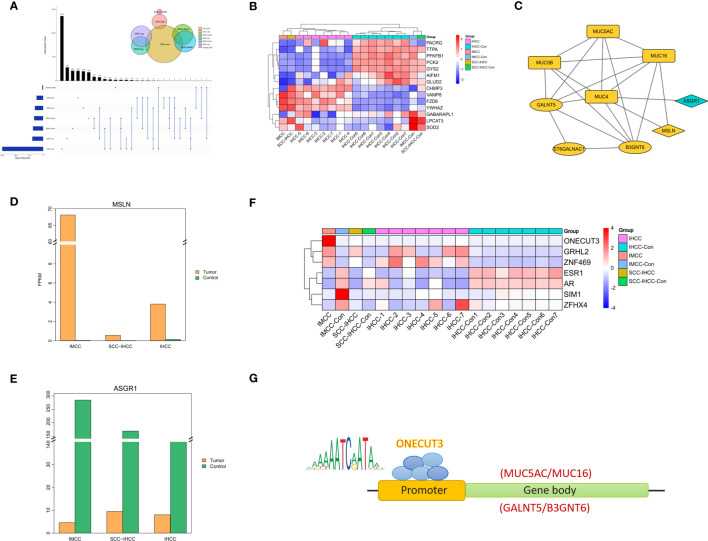
Multi-omics comprehensive analysis of IMCC showing molecules that changed together in genes, RNA and protein levels **(A)**. The heatmap shows the expression of cell death-related genes in IMCC, IHCC and SCC-IHCC **(B)**. String analysis of the interactions between multi-omics convergent genes and mucin and mucopolysaccharide biosynthesis genes **(C)**. Expression comparison in IMCC, IHCC and SCC-IHCC of MSLN **(D)** and of ASGR1 **(E)**. The heatmap **(F)** shows the transcription factors in IMCC that may regulate the expression of mucin and mucopolysaccharide. JASPAR predicts the genes that ONECUT3 may target **(G)**.

By comprehensively analyzing the multi-omics, we found that there are 196 genes with the same changing trend in IMCC (**Figure 3A**). We supposed that mucus secretion is related to necrosis, so we analyzed the expression of cell death-related genes in different cholangiocarcinoma subtypes. This did not identify specific expression of necrosis-related genes in IMCC (**Figure 3B**). Further screening of molecules that might regulate the expression of mucin-producing genes (MUC4, MUC5AC, MUC5B, MUC16) and genes producing mucopolysaccharides (GALNT5, ST6GALNAC1, B3GNT6) revealed that ASGR1 may regulate the expression of MUC4, while MSLN may regulate the expression of MUC4 and MUC16 (**Figure 3C**). There is currently no relevant report on the interaction between MUC4 and MSLN, but the interaction between MUC16 and MSLN plays an important role in the development of ovarian cancer (14). The binding of MSLN and MUC16 has been shown to induce intercellular adhesion (15). Therefore, we speculate that MUC16 may bind to MSLN to regulate mucins synthesis.

We further analyzed the expression of ASGR1 and MSLN in IMCC, SSC-IHCC and IHCC. This showed that MSLN was specifically highly expressed in IMCC, while ASGR1 was not (**Figures 3D, E**). It is speculated that IMCC may increase mucus secretion by up-regulating MSLN. Transcription factors that control the expression of key genes were also screened. A total of 7 transcription factors were identified in 196 annotated genes and their expression was further analyzed in 3 subtypes of cholangiocarcinoma. The results showed that the expression of ONECUT3 was specifically elevated in IMCC (**Figure 3F**). Using the JASPAR database (http://jaspar.genereg.net/) to predict the target genes of ONECUT3, it was found that the motif of ONECUT3 may target for MUC16, MUC5AC, GALNT5 and B3GNT6 (**Figure 3G**). This suggested that IMCC might increase the synthesis of mucin and mucopolysaccharide by up-regulating ONECUT3.

## Discussion

Previous studies have identified the important role of KRAS in the pathogenesis of IHCC (16, 17). Here we report presence of the G12D mutation of KRAS in an IMCC patient. Our transcriptome analysis revealed that the genes upregulated in IMCC are mainly involved in biosynthesis of mucin and mucopolysaccharide. Study showed that IMCC was positive for MUC-1/MUC-5/MUC-6 and CK7/CK8/CK19 (18, 19). Our transcriptome data are highly consistent with the pathological characteristics of IMCC.

From the data of proteome and metabolome from IMCC, we found vigorous cell proliferation with local inflammation and secretion of large amounts of extracellular matrix in IMCC. Ordonez et al. (2003) proved that one-third of cholangiocarcinomas are MSLN positive. Our results show that MSLN is highly expressed in IMCC. In cancer progression, MSLN is known to bind to the cancer antigen MUC16. Therefore, we speculate that MSLN promotes the occurrence of IMCC by up-regulating the secretion of mucin genes such as MUC16.

Through a multi-omics comprehensive analysis, we found that ONECUT3 is highly expressed in IMCC. In mammals, 3 onecut genes have been described: Onecut1 (Hnf6), Onecut2, and Onecut3 (20); Two of the onecut proteins, Hnf6 and Onecut2 (Oc2), play an essential role in the development of the mammalian intrahepatic biliary system (21). Onecut3 (oc3) is essential for zebrafish intrahepatic biliary development, and that oc3 functions when biliary epithelial cells are first specified, thus suggesting that zebrafish oc3 may be the functional ortholog of mammalian Hnf6 (22). Based on our findings, we speculate that ONECUT3 induces the pathogenesis of IMCC by activating the expression of mucins and mucopolysaccharides. If this can be confirmed, MSLN and ONECUT3 may be suitable biomarkers of IMCC.

## Data availability statement

The raw data supporting the conclusions of this article will be made available by the authors, without undue reservation.

## Ethics statement

The studies involving human participants were reviewed and approved by Ethics Committee of Shunde hospital of Southern Medical University. The patients/participants provided their written informed consent to participate in this study. Written informed consent was obtained from the individual(s) for the publication of any potentially identifiable images or data included in this article.

## Author contributions

XZ, HO and JY designed experiments. XZ and CZ performed study. XZ and QL analyzed the data. JY and WW supervised the study.
