# Supplementary figures and images for: Multi-omics integrated analyzed the origin of intrahepatic mucinous cholangiocarcinoma: a case report
Source: Front Oncol. 2023 Jul 21;13:1175707. doi: 10.3389/fonc.2023.1175707 (PMC10401833; doi:10.3389/fonc.2023.1175707)

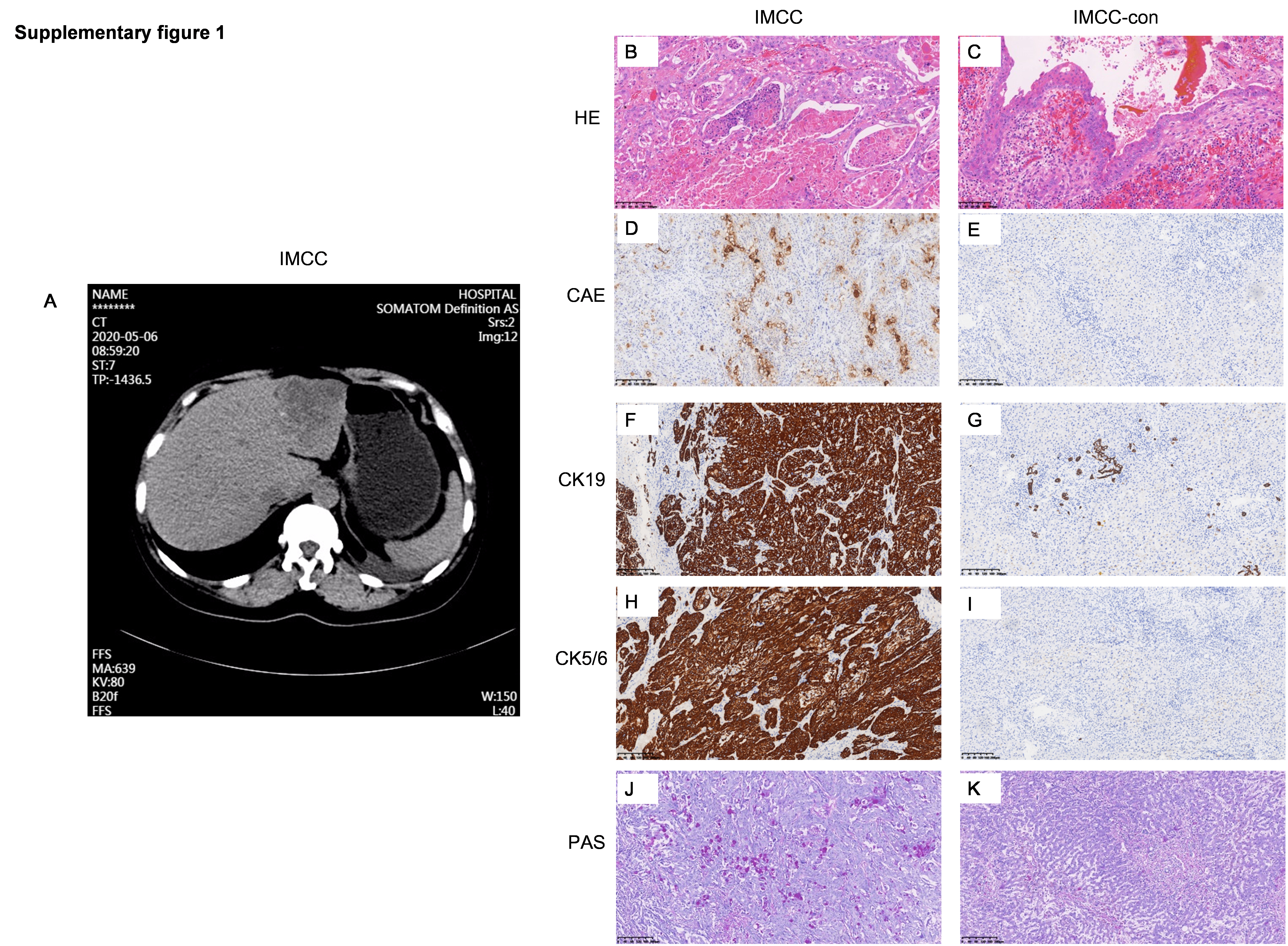

Supplement: Supplementary file 1 [file Image_1.tif]

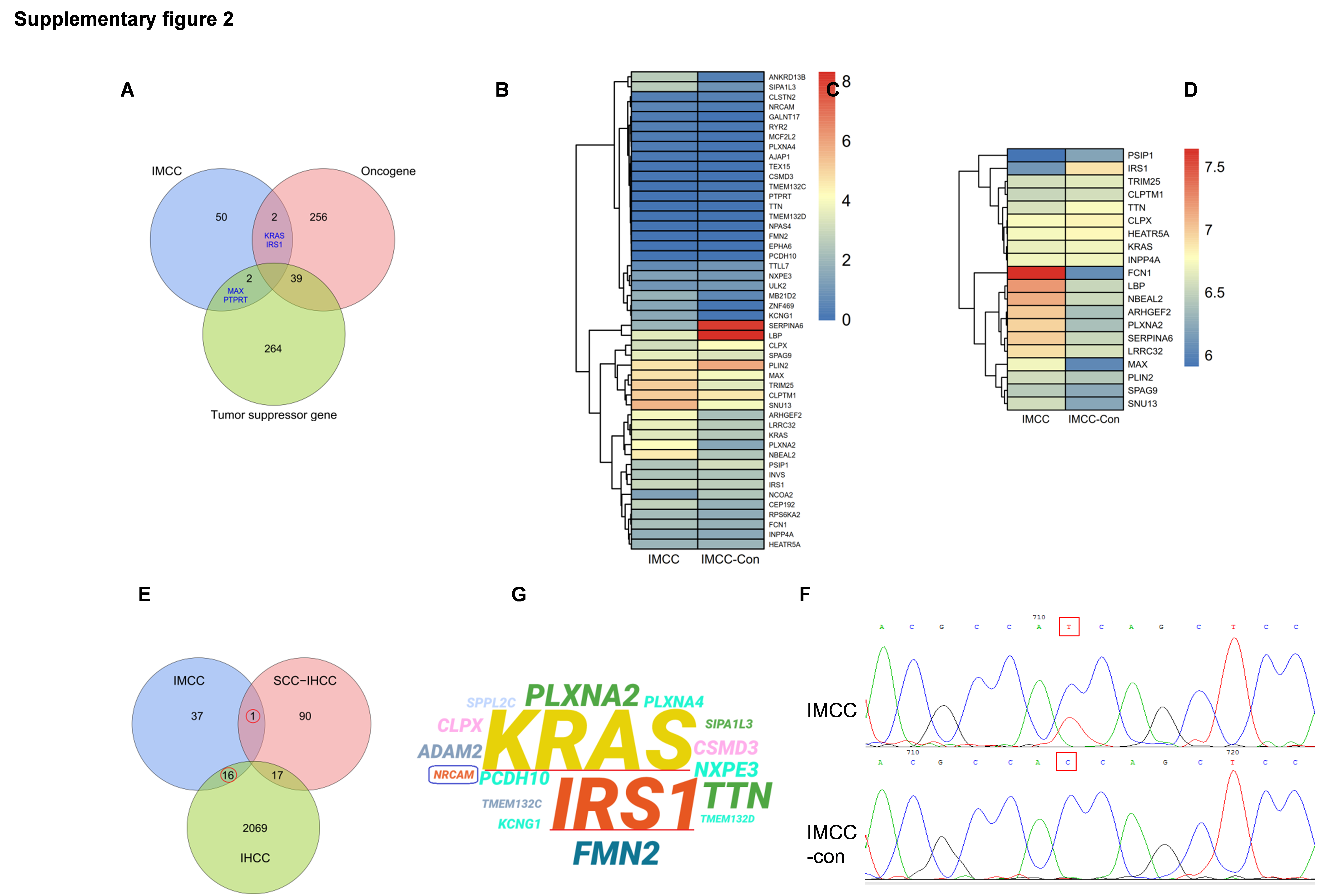

Supplement: Supplementary file 2 [file Image_2.tif]
