# Supplementary material for: Multi-omics integrated analyzed the origin of intrahepatic mucinous cholangiocarcinoma: a case report
Source: Front Oncol. 2023 Jul 21;13:1175707. doi: 10.3389/fonc.2023.1175707 (PMC10401833; doi:10.3389/fonc.2023.1175707)
Supplement: Supplementary file 4 [file DataSheet_1.docx]

**MATERIALS AND METHODS**

**DNA extraction, whole exome sequencing (WES) and somatic mutation analysis**

Genomic DNA was extracted from the frozen IMCC tumor and from adjacent control tissue using the QIAamp DNA Mini Kit (QIAGEN, Cat. No. 51306). DNA quantity and integrity was measured by Nanodrop spectrophotometry (Thermo Fisher Scientific, Inc., Wilmington DE, US) and 1% agarose gel electrophoresis, respectively. Agilent SureSelect Human All Exon v6 (Agilent Technologies, US) was employed to capture DNA samples for library construction. The obtained libraries were sequenced on the Illumina sequencing platform (HiSeq X-10, Illumina, Inc., San Diego, CA, US) to generate 150bp paired-end reads. The entire exome sequencing and analysis was carried out by OE Biotech Co., Ltd. (Shanghai, China). Clean reads were aligned to the reference human_glk_v37 based on 1000 genomes utilizing the Burrows-Wheeler Aligner (BWA, version 0.7.12)[[17](#_ENREF_17)]. The MuTect2[[18](#_ENREF_18)] somatic variant caller was used to determine somatic single-nucleotide variants (SNV) and somatic indel calling. Multiple annotation databases including Refseq, 1000 Genomes, the Catalogue of Somatic Mutations in Cancer (COSMIC) and Online Mendelian Inheritance In Man (OMIM) were referred to for single-nucleotide polymorphism SNP and indel calling. Annotated was performed using ANNOVAR[[19](#_ENREF_19)]. The information on the detected genomic variation was visualized using a Circos diagram.

**Copy number variation (CNV) microarray**

The Affymetrix cytoscan CNV microarray was used to characterize copy number variation. After genomic DNA was extracted and quantified, the samples were labeled, chip hybridized and washed according to the manufacturer's recommendations. The chromosome Analysis Kit (ChAS version 3.1.1.27) was used for raw data analysis and for analysis of copy number variations of each sample. Finally, Nexus Express software (version 3.1, BioDiscovery) was employed to detect differential copy number variation.

**RNA sequencing and gene expression profiling**

Total RNA was extracted from the frozen IMCC tumor and adjacent control tissue using the mirVanaTM RNA Isolation Kit (Applied Biosystems). The Agilent 2100 bioanalyzer (Agilent Technologies, Santa Clara, CA, US) was used to assess RNA integrity. The TruSeq Stranded mRNA LT sample preparation kit (Illumina, San Diego, CA, US) was used to construct libraries that were sequenced on the Illumina HiSeq XTen platform. First, the software tool Trimmomatic was used to process the raw data in fastq format (raw reading)[[20](#_ENREF_20)], and to remove low-quality reads. HISAT2[[21](#_ENREF_21)] was then employed to map clean reads to the human genome (GRCh38.p12). Cufflinks[[22](#_ENREF_22)] was used to calculate the fragments per kilobase per million mapped reads (FPKM) [[23](#_ENREF_23)] of each gene, and the read count of each gene was obtained through HTSeq-counts [[24](#_ENREF_24)]. DESeq (2012) within the R software package [[25](#_ENREF_25)] was used for differential expression analysis. P values <0.05 and fold changes >2 or <0.5 were used as the thresholds for significant differential expression. Enrichment analysis based on Gene Onthology (GO) and Kyoto Encyclopedia of Genes and Genomes (KEGG pathway)[[26](#_ENREF_26)] characteristics of differentially expressed genes (DEG) was performed using R, based on a hypergeometric distribution. Protein interactions were analyzed using the database String (https://string-db.org/), and Cytoscape software was employed to obtain a network map. Standard gene set enrichment analysis (GSEA) was performed with the software provided by the Massachusetts Institute of Technology[[27](#_ENREF_27)]. Normalized enrichment score (NES) and false discovery rate (FDR) were used to quantify enrichment magnitude and statistical significance, respectively.

**Proteome detection and protein expression profiling**

The proteins in IMCC tumor and control tissue were extracted by the ultrasonic lysis method, and protein concentrations were determined using the bicinchoninic acid BCA assay. Subsequently, 100 μg of protein was trypsinized at 37°C for 12 hours. Tandem mass tags (TMT) were attached using the TMT Composite kit (Thermo Fisher Scientific, San Jose, California, US). The labeled peptides were separated and lyophilized using High Performance Liquid Chromatography (HPLC, Agilent 1100). All analyses were performed by a Q-Exactive HF mass spectrometer equipped with a Nanospray Flex light source ((Thermo Fisher Scientific, San Jose, CA, US). Proteome Discoverer software (version 2.4; Thermo Fisher Scientific) was used to identify peptides and proteins through the Sequest HT search engine. The R software package was used for bioinformatic analysis of differentially expressed proteins.

**Metabolome mass spectrometry**

Non-target metabolomics analysis was performed using a 7890B gas chromatograph equipped with a 5977A mass spectrometer (GC-MS, Agilent Technologies, Santa Clara, CA, US) and LC-MS analysis was performed using a Q-Exactive mass spectrometer (Thermo Fisher Scientific, CA, US) equipped with a Nanospray Flex ion source. Metabolites identified by GC-MS were annotated using the LUG database (a non-target database from Lumingbio's GC-MS), and metabolites identified by LC-MS are annotated by progenesis QI (Waters Corporation, Milford, USA). The software used is based on public databases, including http://www.hmdb.ca/http://www.lipidmaps.org/, and https://metlin.scripps.edu, complemented with self-built databases. Differential metabolites were selected based on a combination of the statistical significance threshold of the variable impact (VIP) value on the projection obtained from the orthogonal partial least squares discriminant analysis (OPLS-DA) model and the P-value derived from the two-tailed student *t*-test. Those metabolites with VIP>1.0 and P-value<0.05 were considered to represent a significant difference. The KEGG database was used to identify the pathway of the metabolites.
